# Reduced Flexibility Associated with Metabolic Syndrome in Community-Dwelling Elders

**DOI:** 10.1371/journal.pone.0117167

**Published:** 2015-01-23

**Authors:** Ke-Vin Chang, Chen-Yu Hung, Chia-Ming Li, Yu-Hung Lin, Tyng-Guey Wang, Keh-Sung Tsai, Der-Sheng Han

**Affiliations:** 1 Department of Physical Medicine and Rehabilitation, National Taiwan University Hospital, Bei-Hu Branch and National Taiwan University College of Medicine, Taipei, Taiwan; 2 Department of Physical Medicine and Rehabilitation, National Taiwan University Hospital, Chu-Tung Branch, Hsinchu, Taiwan; 3 Department of Family Medicine, National Taiwan University Hospital, Bei-Hu Branch and National Taiwan University College of Medicine, Taipei, Taiwan; 4 Department of Social Work, National Taiwan University Hospital, Bei-Hu Branch, Taipei, Taiwan; 5 Department of Physical Medicine and Rehabilitation, National Taiwan University Hospital and National Taiwan University College of Medicine, Taipei, Taiwan; 6 Department of Internal Medicine, National Taiwan University Hospital, Bei-Hu Branch and National Taiwan University College of Medicine, Taipei, Taiwan; Rutgers University -New Jersey Medical School, UNITED STATES

## Abstract

**Background:**

The ageing process may lead to reductions in physical fitness, a known risk factor in the development of metabolic syndrome. The purpose of the current study was to evaluate cross-sectional and combined associations of metabolic syndrome with body composition and physical fitness in a community based geriatric population.

**Methods:**

A total of 628 community-dwelling elders attending a geriatric health examination were enrolled in the study. The diagnosis of metabolic syndrome was based on the modified National Cholesterol Education Program Adult Treatment Panel III (NCEP ATP III) criterion with Asian cutoff of waist girth was adopted in this study. Body composition was obtained using bioimpedance analysis, and physical fitness was evaluated through the measurement of muscle strength (handgrip force), lower extremity muscle endurance (sit-to-stand test), flexibility (sit-and-reach test), and cardiorespiratory endurance (2-minute step test). Multivariable logistic regression and correlation analysis were performed to determine the association of metabolic syndrome with body composition and functionality variables.

**Results:**

Metabolic syndrome was associated with increased skeletal muscle index (SMI) (odds ratio (OR), 1.61, 95% confidence interval (CI), 1.25–2.07) and decreased flexibility (OR, 0.97, 95% CI, 0.95–0.99) compared with those without metabolic syndrome. When body mass index was accounted for in the analysis, the association of SMI with metabolic syndrome was reduced. Waist circumference was positively correlated with SMI but negatively correlated with flexibility, whereas high density lipoprotein was positively correlated with flexibility but negatively correlated with SMI.

**Conclusion:**

Reduced flexibility was positively associated with metabolic syndrome independent of age, gender, body composition, and functionality measurements in a community based geriatric population. Significant associations between metabolic syndrome with muscle strength and cardiorespiratory fitness in the elderly were not observed. Furthermore, flexibility should be included in the complete evaluation for metabolic syndrome.

## Introduction

Metabolic syndrome is a combination of several metabolic disorders encompassing visceral obesity, insulin resistance, dyslipidemia, and hypertension [1]. Metabolic syndrome potentiates inflammation and oxidative stress and increases the risk of cardiovascular disease, type II diabetes mellitus, Parkinson’s disease, and various cancers [2–4]. The ageing process is associated with the accumulation of body fat mass and the loss of skeletal muscle mass. The changes in body composition are associated with sedentary behavior and physical inactivity in the elderly, both of which account for the development of metabolic syndrome [5].

Previous research has demonstrated that patients with metabolic syndrome also report lower levels of physical activity, poorer cardiorespiratory endurance, and decreased muscular strength; however, significant variation existed between groups based on gender, age, and social economic backgrounds [6–8]. In elderly populations with metabolic syndrome, lower functional capacity, muscular strength, and flexibility have been reported [8–10]; whereas, other studies reported a lack of association between functionality variables and metabolic syndrome in the elderly [11]. Regarding similar studies using a large sample, Jurca et al. recruited 8570 participants with ages ranging from 20 to 75 years, and found a negative association between leg strength and prevalence of metabolic syndrome, even after adjustment for cardiorespiratory fitness [6]. Wijndaele et al. confirmed the above-mentioned findings by recruiting 1019 Flemish adults aged between 18 to 75 years [8]. Inconsistencies may be due to different sample sizes, variability in the tools used to measure physical fitness, and the use/non-use of multivariable analysis to adjust the confounders. To the best of our knowledge, few studies have investigated the relationship between metabolic syndrome and health-related physical fitness in urban senior citizens, and specifically the influence of body composition [11, 12]. We hypothesized that metabolic syndrome was associated a certain component of health-related physical fitness in community-dwelling elders. Therefore, the aim of the current study was to compare cross-sectional relative and combined associations of metabolic syndrome with body compositions, flexibility, muscle strength and cardiorespiratory fitness in a community based geriatric population.

## Materials and Methods

Enrolled participants were aged greater than 65 years and attended a geriatric health examination between January 2012 and September 2012. The study was approved by the institutional review board of the National Taiwan University Hospital (201201045RIC) and all participants provided their written informed consent in this study. Participants with a history of malignancy, cerebral vascular disorders, neurodegenerative diseases, end stage renal disease, and severe bony deformity were excluded. In addition to standard measurements for anthropometric parameters, serum biochemistry and physical fitness were also evaluated. A self-report questionnaire was used to evaluate leisure and physical activities [13, 14]. Physical activity was quantified using 7 items, each of which had a maximal score of 3 (the most frequent) and a least score of 0 (the least frequent), assessing the frequency of participation in hiking, dancing, swimming, travelling, shopping, martial arts, and gymnastics.

### Diagnosis of metabolic syndrome

Waist circumference was measured using a soft tape measure midway between the 12^th^ rib and the iliac crest with participants in standing. Body mass index (BMI) was calculated as weight (kg) divided by the square of participant height (m). Systolic and diastolic blood pressures were monitored using a standard sphygmomanometer following at least 10 minutes of rest in a seated position. A venous blood sample was drawn after an overnight fast for a measurement of plasma glucose, total cholesterol, low-density lipoprotein (LDL) cholesterol, high-density lipoprotein (HDL) cholesterol, and triglycerides using an auto-analyzer (Hitachi 7250 Special, Hitachi, Tokyo, Japan).

The modified National Cholesterol Education Program Adult Treatment Panel III (NCEP ATP III) criterion with Asian cutoff of waist girth was adopted in this study [15]. The diagnosis of metabolic syndrome was based on the presence of three or more of the following criteria: (1) central obesity with a waist circumference greater than 90 cm in males, and 80 cm in females; (2) systolic blood pressure higher than 130 mmHg or diastolic blood pressure higher than 85 mmHg, or previously diagnosed hypertension; (3) HDL cholesterol lower than 40 mg/dl in males, and 50 mg/dl in females; (4) fasting glucose higher than 100 mg/dl, or previously diagnosed type 2 diabetes; (5) triglyceride higher than 150 mg/dl [16].

### Measurement of health related physical fitness

**Muscular strength**. The maximal grip force of the dominant hand was measured to represent upper extremity muscular strength using an analog isometric dynamometer (Baseline hydraulic hand dynamometer, Fabrication Enterprises Inc., Irvington, NY, USA)–the highest value of three attempts was used for analysis [17]. Lower extremity muscular endurance was evaluated using a sit-to-stand test (participants were asked to stand and sit repeatedly for 30 seconds [with their arms folded across their chests]; the number of repetitions was recorded) [10, 18]. The above mentioned equipment and protocol were shown to have good test-retest strength reliability according to a previous study [19].

**Cardiorespiratory endurance**. Cardiorespiratory endurance was evaluated using a timed step test. Participants were asked to alternately raise each knee to the midway between the patella and iliac crest for 2 minutes; the number of repetitions was recorded [18, 20]

**Flexibility**. Flexibility was measured using a modified sit-and-reach test. Participants were seated at the front edge of a chair, with both legs extended forward with their feet flat on the floor. With their hands on top of each other and arms outstretched, the participant was asked to reach as far forward as possible toward their toes. The distance between the tip of the middle finger and toes was recorded. A negative score indicated the failure of the finger tips to touch the toes, whereas a positive value demonstrated the flexibility to reach past the toes [18].

**Body composition**. A multi-frequency bioelectrical impedance machine (InBody 720, Biospace, Seoul, Korea) was used to estimate arthropometric parameters. All the body composition data were obtained in the device by using the manufacturer’s proprietary software. The cross-validation of this instrument had been tested by Kim & Kim and demonstrated acceptable accuracy for the estimation of body composition in the elderly [21]. An 8-point tactile electrode system was used to measure the total and segmental impedance and phase angle of alternating electric current at four different frequencies (5 kHz, 50 kHz, 250 kHz, and 500 kHz). During data measurement, the participants were asked to stand upright with their feet placed on foot electrodes on the device platform with their arms away from their torso. Participants were asked to grasp handles for the device. All points of contact contained electrodes. All subjects were instructed to fast and avoid exercise 8 hours before bioelectrical impedance measurements. The calculation of skeletal muscle mass (SMM) and peripheral body fat mass (PBFM) were evaluated. Relative ratios of PBFM to body weight were PBFM%, whereas skeletal muscle index (SMI) referred to SMM divided by the square of the height in meters [22]. All the measurements were performed in the air-conditioned environment with the temperature of about 28 degrees Celsius. The participants were not refrained from caffeine ingestion 8 hours before the tests.

### Statistical Analysis

All the participants were divided into two groups: those presenting with metabolic syndrome and those without. Based on two previous studies with similar designs, we assumed the prevalence of one examined factor was 12% in the metabolic syndrome group and 6% in the reference group. The sampling ratio of the metabolic syndrome group to the reference group was 0.5. Under the alpha value of 0.05 and the beta value of 0.2, the sample size was estimated to be 576 in total [11, 12].

We reported continuous variables as the mean and standard deviation and categorical variables as n (%). Continuous and categorical variables were compared using a Student *t*-test and chi-square test, respectively. Since we divided our participants into two groups based on the presence of metabolic syndrome, the outcome variable was considered binary and the logistic regression was suitable for estimating the probability of having metabolic syndrome compared with that of being participants without metabolic syndrome. The relationship between the components of metabolic syndrome and the parameters of anthropometry, serum biochemistry and physical fitness were examined using a univariate and multivariate logistic regression and expressed by odds ratios (ORs) and 95% confidence intervals (CIs) [23]. The ORs indicated the odds of having metabolic syndrome given an independent variable divided by the odds of having metabolic syndrome without the explanatory variable. The receiver operating characteristic (ROC) curves were constructed to assess the ability of each physical fitness parameter to discriminate between groups with or without metabolic syndrome. An area under the curve (AUC) of 0.5 was indicative of no discriminative value of the selected variable. A Spearman correlation coefficient was used to determine if a correlation existed between parameters of physical fitness and components of metabolic syndrome and the assumption of normality of the examined variables was not required while conducting the analysis. All statistical analyses were performed using SAS software (Version 9.2, SAS Institute, Cary, NC, USA)with a 5% significance level (P<0.05).

## Results

A total of 628 participants were enrolled (mean age: 72.2±5.3 years; range: 65–100 years), 48.5% of participants were males. The prevalence of metabolic syndrome was 28.0%. Those participants with metabolic syndrome were on average older, more likely to be female, have lower high-density lipoprotein cholesterol, higher body weight, higher body mass index, waist circumference, blood pressure, fasting plasma glucose, and triglycerides. Regarding the performance of physical fitness, those with metabolic syndrome had less flexibility, slower gait speeds, and higher physical activity scores, skeletal muscle mass (SMM), skeletal muscle index (SMI), peripheral body fat mass (PBFM) and PBFM% (Table 1).

**Table 1:** Baseline characteristics of elderly adults with and without metabolic syndrome.

**Variable**	**Total sample**	**No metabolic syndrome**	**Metabolic syndrome**	**P value**
	**N = 628**	**n = 452**	**n = 176**	
Age (year±SD)	72.2±5.3	72.1±5.4	72.5±5.1	0.01*
Gender (male, %)	48.5%	51.3%	41.4%	0.006*
**Anthropometry measurement**				
Body weight, kg	60.4±9.6	58.9±9.2	64.1±9.4	<0.001*
Body height, cm	159.0±8.0	159.3±8.0	158.3±7.8	0.78
Body mass index, kg/m2	23.8±2.9	23.1±2.7	25.5±2.6	<0.001*
Waist circumference, cm	82.8±8.0	80.9±7.7	87.7±6.7	<0.001*
**Hemodynamic measurement**				
Systolic blood pressure, mmHg	129.8±17.2	127.1±16.8	136.9±16.5	<0.001*
Diastolic Blood Pressure, mmHg	70.2±10.2	69.6±9.6	71.8±11.2	0.001*
**Biochemical measurement**				
Fasting plasma glucose, mg/dL	100.0±18.7	96.1±15.7	110.2±21.8	<0.001*
Total cholesterol, mg/dL	185.1±31.0	186.5±31.8	181.8±28.8	0.14
Triglyceride, mg/dL	120.4±67.3	102.4±45.6	166.8±88.7	<0.001*
High-density lipoprotein cholesterol, mg/dl	51.0±12.8	54.4±12.5	42.0±8.6	<0.001*
Low-density lipoprotein cholesterol, mg/dl	113.1±26.1	113.1±26.6	113.0±24.9	0.76
**Physical activity**				
Physical components of questionnaires, points	5.8±3.1	5.8±2.9	5.9±3.4	0.002*
**Body composition**				
Skeletal muscle mass, kg	22.8±5.2	22.7±5.2	23.2±5.3	0.005*
Skeletal muscle index, kg/m2	8.9±1.5	8.8±1.5	9.1±1.7	<0.001*
Peripheral body fat mass, kg	18.4±5.5	17.1±5.2	21.6±4.9	<0.001*
Peripheral body fat mass%, %	30.4±7.2	29.1±7.3	33.8±5.8	<0.001*
**Functional capacity**				
Flexibility (sit and reach), cm	-0.2±13.4	0.7±13.7	-2.5±12.4	0.04*
Grip strength, kg	26.9±15.3	27.6±17.3	25.3±8.3	0.29
Relative grip strength (grip strength/body weight), %	44±25	46±28	39±10	<0.001*
30-second chair stand test, number	15.0±5.0	15.3±5.1	14.4±4.5	0.80
Cardiorespiratory endurance (2-minute step test), number	87.4±19.8	88.0±19.8	86.1±20.0	0.55
Gait speed, m/s	1.1±0.2	1.1±0.2	1.0±0.2	<0.001*

* indicated P<0.05.

A prior analysis was performed to determine which variable of body compositions would be selected into the final logistic regression model. Although SMI, PBFM, and PBFM% were associated with metabolic syndrome using a univariate analysis, only SMI had a significant positive association with metabolic syndrome in consideration of other parameters of body compositions (Table 2).

**Table 2:** The association between metabolic syndrome and body composition^a^.

	**Univariate**		**Multivariate**	
	**OR**	**95% CI**	**OR**	**95% CI**
Skeletal muscle mass, kg	1.01	0.98, 1.05	1.00	0.80, 1.25
Skeletal muscle index, kg/m^2^	1.13*	1.01, 1.29	1.99*	1.32, 2.99
Peripheral body fat mass (PBFM), kg	1.18*	1.13, 1.22	1.00	0.79, 1.27
PBFM%, %	1.10*	1.07, 1.13	1.04	0.88, 1.22

* indicated p<0.05.

^a^ The univariate analysis disclosed the crude associations of metabolic syndrome with each parameter, whereas the multivariate analysis reported the adjusted values by including all the measurements of body composition in the regression model. The associations were represented by the odds ratio (OR) and 95% confidence intervals (CI).

The logistic regression models (unadjusted and adjusted) for age, gender, intensity of physical activity, and BMI are presented in Table 3. Female gender was a strong predictor of metabolic syndrome alone or in combination with other parameters (model 1 to 5). The presence of metabolic syndrome was associated with an increase in SMI (OR = 1.61, 95% CI = 1.25–2.07) and a decrease in flexibility (OR = 0.97, 95% CI = 0.95–0.99) after adjustment for age and gender (model 2). The addition of “intensity of physical activity” in the model did not mitigate the significant association of SMI and flexibility with metabolic syndrome (model 3). The “volume of physical activity” was classified as being high or low intensity using the median of the summed scores as the reference point. Lower physical activity was not associated with metabolic syndrome in the present population. To further examine whether BMI confounded the association of metabolic syndrome with physical fitness and body composition, subsequent analyses were performed that including BMI (in model 4) in which the association of SMI and metabolic syndrome became insignificant.

**Table 3:** The association of metabolic syndrome with measurements of body composition and physical fitness^a^.

	**Model 1**		**Model 2**		**Model 3**		**Model 4**		**Model 5**	
	**OR**	**95% CI**	**OR**	**95% CI**	**OR**	**95% CI**	**OR**	**95% CI**	**OR**	**95% CI**
Age, year	1.02	0.98, 1.05	1.00	0.96, 1.04	1.00	0.96, 1.04	1.01	0.97, 1.06	1.02	0.97, 1.06
Gender(reference: female)	0.64*	0.45, 0.92	0.26*	0.13, 0.53	0.26*	0.13, 0.53	0.36*	0.18, 0.68	0.38*	0.20, 0.70
Skeletal mass index, kg/m2	-	-	1.61*	1.25, 2.07	1.61*	1.25, 2.07	1.05	0.91, 1.22	1.06	0.91, 1.22
Flexibility (sit and reach), cm	-	-	0.97*	0.95, 0.99	0.97*	0.95, 0.99	0.97*	0.95, 0.99	0.97*	0.95, 0.99
Grip strength, kg	-	-	0.98	0.95, 1.02	0.98	0.95, 1.02	0.99	0.96, 1.02	-	-
Relative grip strength (grip strength/body weight)	-	-	-	-	-	-	-	-	0.24	0.02, 3.14
30-second chair stand test, number	-	-	0.98	0.94, 1.02	0.98	0.94, 1.02	1.00	0.95, 1.05	1.00	0.95, 1.05
2-minute step test, number	-	-	1.00	0.98, 1.01	1.00	0.98, 1.01	0.99	0.98, 1.00	0.99	0.98, 1.01
Gait speed, m/s	-	-	0.62	0.23, 1.66	0.62	0.23, 1.66	0.89	0.31, 2.51	0.92	0.33, 2.60
High physical activity versus Low physical activity (reference)	-	-	-	-	1.01	0.69, 1.49	1.12	0.74, 1.69	1.05	0.98, 1.12
Body mass index, kg/m^2^	-	-	-	-	-	-	1.38*	1.27, 1.49	1.35	1.23, 1.47

* indicated p<0.05.

^a^ The associations were represented by odds ratio and 95% confidence intervals (CIs).

**Figure 1:**
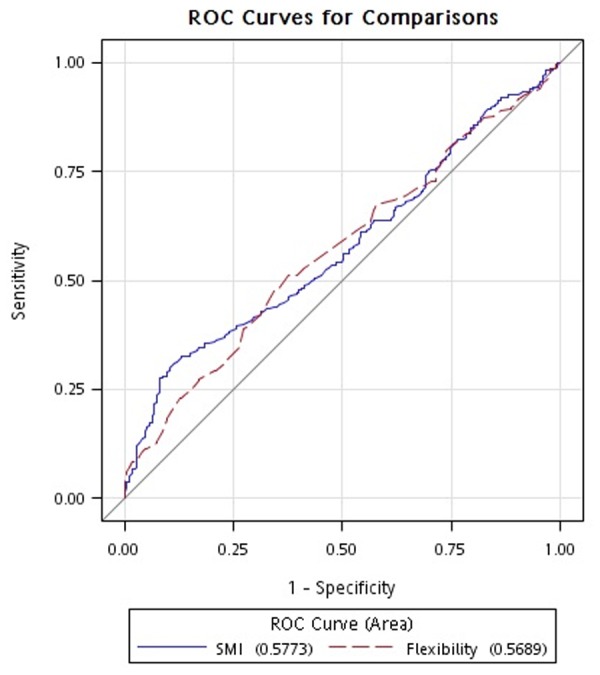
Receiver operating characteristics (ROC) curve of skeletal muscle index (SMI) and flexibility in discriminating the presence of metabolic syndrome. An area under the curve (AUC) of 0.5 was indicative of no discriminative value of the selected variable.

The capability of each physical fitness-associated parameter to discriminate the absence or presence of metabolic syndrome is presented in Table 4. The receiver operation curve analysis indicated that only the AUC of SMI and flexibility had lower limits of 95% CI exceeding 0.5 (Fig. 1). The correlation between physical fitness-associated parameters used to define metabolic syndrome are presented in Table 5. Results indicate a positive correlation between SMI with waist circumference and a negative correlation with high-density lipoprotein cholesterol. Whereas, flexibility was negatively correlated with waist circumference and positively correlated with high-density lipoprotein cholesterol.

**Table 4:** Body composition and physical fitness to discriminate for the presence of metabolic syndrome.

	**Area under curve**	**95%CI**	**P value**
Skeletal muscle index	0.57	0.52–0.62	<0.001*
Flexibility (sit and reach test)	0.57	0.52–0.62	0.007*
Grip strength	0.55	0.50–0.60	0.146
30-second chair stand test	0.56	0.50–0.60	0.04*
Cardiorespiratory endurance (2-minute step test)	0.52	0.47–0.57	0.17
Gait speed	0.54	0.49–0.59	0.89

* indicated p<0.05.

**Table 5:** Correlation of physical fitness associated parameters with components of metabolic syndrome^a^.

	**WC**	**SBP**	**DBP**	**FPG**	**HDL**	**TG**
Skeletal muscle index	0.41* (<0.001)	0.07 (0.07)	-0.05 (0.18)	0.06 (0.13)	-0.25* (<0.001)	0.05 (0.15)
Flexibility (sit and reach test)	-0.27* (<0.001)	-0.05 (0.18)	0.04 (0.25)	-0.05 (0.19)	0.20* (<0.001)	-0.07 (0.05)
Grip strength	0.13* (<0.001)	0.05 (0.25)	-0.02 (0.67)	-0.08 (0.83)	-0.08* (0.03)	-0.03 (0.39)
30-second chair stand test	-0.09* (0.02)	-0.01 (0.79)	-0.01 (0.79)	0.0002 (0.99)	0.05 (0.16)	-0.07 (0.05)
Cardiorespiratory endurance (2-minute step test)	-0.03 (0.34)	0.03 (0.32)	0.03 (0.32)	-0.019 (0.63)	0.04 (0.31)	-0.06 (0.10)
Gait speed	-0.07 (0.07)	-0.03 (0.32)	-0.04 (0.32)	-0.05 (0.17)	0.05 (0.14)	-0.03 (0.39)

Abbreviation: WC, waist circumference; SBP, systolic blood pressure; DBP, diastolic blood pressure; FPG, fasting blood plasma; HDL, High-density lipoprotein; TG: triglyceride;

* indicated p<0.05.

^a^ The values were expressed by correlation coefficients and p value in brackets.

## Discussion

The current study investigated the association between components of metabolic syndrome and physical fitness measurements in a geriatric population. Results demonstrated that the presence of metabolic syndrome was associated with an increase in SMI, although the significance of the association with SMI was diminished when BMI was added as part of the regression model. Compared with previous studies, the most novel finding was the positive association between the presence of metabolic syndrome and a decrease in flexibility. Our correlation analysis also indicated that flexibility was positively correlated with high-density lipoprotein but negatively associated with waist circumference.

Skeletal muscles play a crucial role in regulating glucose utilization, dysfunction of which leads to hyperglycemia, one of the five components of metabolic syndrome [24]. The aging process is associated with skeletal muscle atrophy and fat deposition, which was reported to be associated with insulin resistance in clinical observational studies [25]. It has been suggested that the presence of metabolic syndrome is associated with lower SMM, although our research indicated a positive association of SMM and SMI in participants with metabolic syndrome. Among the parameters of body composition, SMI was the most highly associated, likely mediated by BMI because the statistical significance of SMI was lost by including BMI in the regression model. However, in the univariate analysis, the metabolic syndrome group had higher PBFM and its corresponding percentage to the body weight, suggesting greater fat infiltration in participants with metabolic syndrome. However, compared with BMI, our findings suggest that fatty infiltration of skeletal muscle in the elderly is less significantly associated with the development of metabolic syndrome.

Reduced flexibility was found to be associated with the presence of metabolic syndrome and the observed association was independent from age, gender, and BMI. In the present study, flexibility, quantified by the ability of participants to reach toward their toes while in a sitting position, encompassed the bendability of lumbar spine, hip joint, and hamstrings [26, 27]. The inverse association between flexibility and metabolic syndrome has never been reported in previous literature investigating similar populations, most of which focused on muscle strength and cardiorespiratory endurance [11, 12]. To clarify which component of metabolic syndrome contributed most to lower body flexibility, a correlation analysis was performed between the variables of flexibility and waist circumference, systolic blood pressure, diastolic blood pressure, fasting blood plasma, high-density lipoprotein, and triglycerides. Among the six measurements related to the diagnosis of metabolic syndrome, waist circumference possessed the strongest negative correlation with flexibility (Table 5). Therefore, we speculated that an increase in waist circumference hampered the ability to forward reach due to trunk restriction, resulting in decreased flexibility in participants with metabolic syndrome.

Associations between metabolic syndrome and cardiorespiratory fitness were reported in previous studies but were not identified in the current results [28–30]. Two reasons might account for this finding. First, prior research defined cardiorespiratory fitness as the maximal amount of oxygen consumed during high intensity exercise, most of which were calculated from an equation consisting of results from a walk or shuttle run test, and participant age, gender, and body weight [28–30]. Hence, the association reported in previous literature may be due in part from demographic differences between participants with and without metabolic syndrome. Second, the average age of the current participants was 72.2 years, which is higher than that in studies reporting an inverse association between cardiorespiratory fitness and metabolic syndrome [28–32]. Aging is known to decrease stride length and gait speed, both of which are major determinants of the walk test performance [33]. Therefore, the differences observed in cardiorespiratory fitness between elders with and without metabolic syndrome might become too small to be clinically significant in an advanced age population by using gait speed and step numbers as surrogate indicators.

Metabolic syndrome leads to a pro-inflammatory state and pro-inflammatory cytokine release from adipose tissues may potentiate skeletal muscle catabolism [34]. An association between metabolic syndrome and lower body muscle strength has been reported in several similar studies investigating adult participants with a wide age range [6–8, 10]. In a previously reported case-control study, the univariate analysis indicated that elderly women with metabolic syndrome had reduced relative muscle strength in their lower extremities [10]. However, the current results did not reveal a significant association between metabolic syndrome and muscular strength in an elderly population. The cause of absence in association might be that the current study used maximal grip force (a type of isometric strength test) and numbers of full stands from sitting to measure muscle strength of upper extremities and endurance of lower extremities, respectively, which differed from measurements using resistance adjustable machines in prior research.

There are some limitations in this study. First, this study employed a cross-sectional design, thereby the causal relationship of change in physical fitness and development of metabolic syndrome could not be explored. Second, the amount of physical activity was estimated through a set of questionnaires evaluating the frequency of participation in sport and outdoor activities, the exact values of which could not be compared with other studies using different measurements. Therefore, physical activity was dichotomized into lower and higher levels by its median score and was only used to adjust other variables in the regression model. Third, the participants were urban civilians volunteering for an annual health examination and whether our results could be generalized to the overall old population required further studies covering elders with different demographic and social economic backgrounds is unclear.

## Conclusions

The current study demonstrated that elderly participants with metabolic syndrome had a higher SMI, although the association became less significant when participant BMI was accounted for. Reduced flexibility was positively associated with the presence of metabolic syndrome independent of age, gender, and other measurements regarding body composition and physical fitness. In the future, exercise interventions to increase flexibility should be implemented to test its possible therapeutic effect on metabolic syndrome. Furthermore, flexibility should be included in the complete evaluation for metabolic syndrome.

## Supporting Information

S1 Material Institution review board of this study. (PDF)

S2 Material Characteristics and measurements of physical fitness in elders with and without metabolic syndrome. All the information was anonymized.
(XLS)
