# Supplementary material for: Reduced Flexibility Associated with Metabolic Syndrome in Community-Dwelling Elders
Source: PLoS One. 2015 Jan 23;10(1):e0117167. doi: 10.1371/journal.pone.0117167 (PMC4304714; doi:10.1371/journal.pone.0117167)
Supplement: S1 Material — (PDF) [file pone.0117167.s001.pdf]

發文方式：郵寄

檔　　號：
保存年限：

國立臺灣大學醫學院附設醫院　函

地址：100臺北市中山南路7號
承辦人：魏麗軒
電話：02-2312-3456轉63595
傳真：02-2395-1950
電子信箱：ntuhrec@ntuh.gov.tw

108
台北市萬華區康定路37號

受文者：國立台灣大學醫學院附設醫院北護分院內科部蔡克嵩醫師

發文日期：中華民國101年2月8日
發文字號：校附醫倫字第1013700436號
速別：普通件
密等及解密條件或保密期限：普通
附件：如文

裝

主旨：有關　台端所主持之「社區老人體適能常模之建立/The establishment of norms for physical fitness in the community- dwelling elderly.」（本院案號：201201045RIC）純學術臨床試驗案，符合簡易審查條件及研究倫理規範，通過本院研究倫理委員會審查，同意核備，並提C研究倫理委員會第26次會議報備追認，請　查照。

訂

說明：

一、本臨床試驗核准之有效期限為1年，計畫主持人應於到期前3個月至6週向本會提出持續審查申請表，本案需經持續審查，方可繼續執行。

二、本臨床試驗計畫若需變更、暫停執行、中途終止或結束時，主持人應向本會提出審查申請。

線

三、本會同意之文件版本日期如下：

(一)臨床試驗計畫書：2011-1-13。

(二)受試者說明及同意書：2012-1-13。

(三)問卷：2012-1-13。

四、臨床試驗執行期間，請確實依據「人體研究法」、「研究用人體檢體採集與使用注意事項」之相關規定辦理；並請計畫主持醫師保存所有文件備查。

五、依據國際醫學雜誌編輯委員會(The International

擬請轉陳師存查

101. 2. 10

台大北護分第 [illegible] 號

Committee of Medical Journal Editors, ICMJE)之投稿規定，臨床試驗研究計畫投稿者，需於招募第一位受試者參與試驗前，將通過研究倫理委員會審核之臨床試驗計畫資料登錄於臨床試驗公開網站，完成登錄作業後，國際醫學雜誌編輯委員會(ICMJE)才會接受研究結果之發表。WHO對臨床試驗研究計畫之定義為任何對受試者或特定族群進行一個或多個與健康有關的介入措施(如藥物、外科處置、器材、行為治療、飲食介入及照護過程改變)以評估對健康的效益之計畫，非屬上述臨床試驗計畫，請計畫主持人自行決定是否登錄。

六、本院已向美國國家衛生研究院(National Institutes of Health, NIH) ClinicalTrials. gov 網站- Protocol Registration System (PRS https:// register. clinicaltrials.gov/)申請本院專用帳號，供本院計畫主持人(PI) 登錄所主持之臨床試驗研究計畫，登入網頁之帳號及密碼如下列：

(一)Organization：NTaiwanUH

(二)User Name：NTUH

(三)Password：NTUH99

七、隨函檢附臨床研究重要訊息通知單，請依計畫需要辦理相關事宜。

八、受試者同意書須於左上方加註 倫委會案號：201201045RIC始得使用。

正本：國立台灣大學醫學院附設醫院北護分院內科部蔡克嵩醫師
副本：本院研究倫理委員會

院長 陳明豐
